# Injuries in German national short-track speed skating athletes

**DOI:** 10.1016/j.jsampl.2024.100080

**Published:** 2025-01-02

**Authors:** Jan Ziegler, Heidrun Beck, Roland L. Bell, Dominik Matzner, Xinggui Tian, Stefan Zwingenberger

**Affiliations:** a Department of Trauma and Orthopedic Surgery, Sports Traumatology, University Hospital Carl Gustav Carus at Dresden University of Technology, Fetscherstreet 74/2, House 30, 01307 Dresden, Saxony, Germany; b Department of Trauma and Orthopedic Surgery, University Hospital of Coventry and Warwickshire, Clifford Bridge Rd, Coventry, CV2 2DX, United Kingdom; c Independent Researcher, Germany

**Keywords:** Short-track speed skating, Sports injury, Reporting of epidemiological data on injury and illness in sport

## Abstract

**Objectives:**

*Short-Track Speed Skating* (STSS) is a high-intensity ice sport characterized by fast-paced races and proximity among athletes, which inherently poses a risk for falls and injuries. This paper presents a comprehensive analysis of orthopedic-traumatological injuries in German national STSS athletes with comparisons to previous studies.

**Design:**

Retrospective analysis.

**Methods:**

Medical records of 69 athletes (30 females, 39 males) who were part of the German national team between 2009 and 2020 were analyzed retrospectively based on a new standardized injury surveillance system published by the International Olympic Committee in 2020**.**

**Results:**

The average career duration of athletes on the German STSS national team was 10.9 ​± ​4.4 years, with each athlete accumulating 10,116 ​± ​4326 ​h of exposure to risk during training. A total of 371 orthopedic-traumatological injuries requiring medical consultation (IRMCs) were recorded, with an estimated incidence of 0.53 per 1000 ​h of exposure. 69.8 ​% of the observed injuries affected the lower extremities, primarily involving the knee and ankle. Joint injuries were the leading cause of IRMCs, accounting for 16.3 ​% of cases. The majority of IRMCs occurred during on-ice training (45.0 ​%), followed by off-ice training (30.0 ​%), and competitions (25.0 ​%).

**Conclusion:**

This was the first implementation based on a standardized injury surveillance system in STSS. Important information about career-related injuries and injury patterns resulted from the analyses. However, further prospective research is needed to support the prevention of health issues in STSS.

## 1 Introduction

Short-Track Speed Skating (STSS) is an Olympic discipline in which athletes travel at speeds of up to 50 ​km/h around an enclosed track, making it a sport with high injury potential [1]. In addition to the immediate risk of falls and collisions, thousands of training and competition hours at a high degree of exertion pose a risk for sport-specific degenerative musculoskeletal health problems [2]. As in any other sport, a comprehensive understanding of injury patterns and potential risk factors is crucial to secure the physical integrity of athletes thus building the foundation for a successful career in STSS and maintaining physical health after their sports careers.

Previous studies have investigated illnesses and orthopedic injuries in STSS athletes [[1], [2], [3], [4], [5], [6], [7], [8]]. See Supplementary Material Table 1.

Parameters commonly included were the anatomical injury location and injury incidence. The scope of investigations varied, from injuries observed during a single multisport event [4] to periods as long as six years [8]. Injury incidence rates also varied widely depending on the study's reference timeframe. For instance, Brownlow and Mc Caig [3] reported 4.4 injuries per 1000 ​h of exposure, whereas Quinn et al. [1] calculated a probability of 64.2 ​% for STSS athletes to experience at least one injury during a season without knowledge of their actual exposure to risk. Dittmer [2] surveyed 100 STSS athletes over a period of 18 months. According to their findings, an athlete suffers 0.38 injuries per year. As can be seen the previous studies often used individually developed evaluation methods and lacked standardized guidelines for analysis. This hindered not only comparisons within the field of STSS but also impeded broader comparisons with other sports.

To address these issues, the International Olympic Committee (IOC) published an update on recommendations for a standardized epidemiological investigation of illness and injury in sports [9]. The present study aimed to apply these recommendations to a dataset describing German national STSS athletes, thereby setting the foundation for uniform analyses to assess measures implemented to improve safety and reduce injuries in STSS.

## 2 Methods

### 2.1 Study design and setting

This retrospective injury surveillance assessment was conducted between 2019 and 2023 ​at the University Hospital Carl Gustav Carus Dresden, Germany. The study complied with the ethical standards and with the Helsinki Declaration of 1975, as revised in 2008, and was approved by the ethics committee of the Technical University of Dresden (BO-EK-185032021). Informed consent was obtained from the study participants.

### 2.2 Participants

Medical records of 69 STSS athletes (30 females, 39 males), who were members of the German national team were analyzed retrospectively. The inclusion criterion was membership in the German national STSS team. The observation period started with the documented beginning of each athlete's membership in the German national team or their first official race, depending on the information available. The observation period for all athletes ended March 1, 2020, or on the date an athlete left the national team. Three additional athletes could not be included in the observation due to missing medical data.

### 2.3 Data acquisition and variables

The outpatient clinic was the primary point of contact for all medical matters concerning the athletes. Therefore, the data comprised the complete medical records of the STSS athletes. The medical records included patient histories detailing incidents leading to injuries, findings from clinical examinations and diagnostic procedures, as well as records of therapeutic interventions. In addition, the data included records of annual standardized examinations conducted in accordance with the guidelines set by the German Olympic Sports Confederation (DOSB). For this study, all orthopedic and traumatological injuries requiring medical consultation (IRMC) were evaluated. Details on the timing and circumstances of each injury, the affected body part, and the injury type were documented and categorized as outlined in Table 1.

**Table 1:** Classification of injuries requiring medical consultation (IRMC).

Characteristic	Characteristic value
Date of consultation	
Age at the time of consultation	
Number of years in STSS until consultation	
Diagnosis according to ICD-10	
Affected bodyside	Right, left, bilateral, central, unknown
Relationship to sports activity	Direct, indirect, no correlation
Circumstances of the incident leading to consultation	On-ice vs. off-ice; training vs. Competition
Pathomechanism	Acute, repetitive; (see table 1^a^)
Mode of onset	Sudden onset vs. gradual onset
For health issues with sudden onset	Noncontact- vs. contact-injuries Contact- injuries – could result from indirect or direct contact to athlete or object (see table 2^a^)
Affected body regions	Head, neck, shoulder, chest, upper arm, hand, wrist, abdomen, hip/groin, thigh, knee, lower leg, ankle, foot, unspecified, multiple (see table 4^a^)
Involved tissue types	Muscle/tendon, nerves, bone, cartilage/synovium/bursa, ligament/joint capsule, superficial tissue/skin, blood vessels, internal organs, unspecified (see table 5^a^)
Type of health issue	Muscle injury, muscle contusion, compartment syndrome, tendinopathy, tendon rupture, brain and spinal cord injury, peripheral nerve injury, fracture, bone stress injury, bone contusion, avascular necrosis, physis injury, cartilage injury, arthritis, synovitis, bursitis, joint sprain (ligament tear or acute instability episode), chronic instability, superficial contusion, laceration, abrasion, blood vessels, internal organ, nonspecific (see table 5)
Training and competition time lost (“time loss”)	(See table 10^a^)
Initial vs. subsequent injury	Initial injury vs. subsequent injury for subsequent injuries: Recurrence vs. exacerbation

*Note*.

a Referenced tables 1, 2, 4, 5 and 10 are part of the IOC consensus statement on methods for recording and reporting of epidemiological data on injury and illness in sports 2020. *The Orthopaedic Journal of Sports Medicine*, *8*(2), 325967120902908. https://doi.org/10.1177/2325967120902908.

In accordance with definitions and guidelines from the IOC Statement, the following outcome measures were addressed: *multiple injuries*, *subsequent injuries*, *exacerbation*, and *injury severity*. These were operationalized as follows:

In cases where a single incident resulted in *multiple injuries*, such as a knee contusion accompanied by a chin laceration, each injury was documented as a separate IRMC. For severe injuries like fractures, any additional injuries within the same body region (e.g., contusions) were recorded as separate IRMC only if documented as distinct diagnoses in the patient's medical records. *Subsequent injuries* to a previously affected body region were classified either as an *exacerbation*, if recovery from the initial symptoms was incomplete, or as new IRMCs, if full recovery had been achieved. *Injury severity* was quantified by calculating the number of days from the IRMC to full performance recovery.

*Exposure to risk* was determined individually based on each athlete's career duration and their documented weekly hours of training both on and off the ice. Average weekly training hours were recorded annually. To estimate the total exposure to risk, calculations were based on an average of 40 training weeks per year, in line with the annual schedule for German national STSS athletes. Exposure estimates excluded competition periods due to insufficient documentation.

### 2.4 Data analysis

Analyses were performed in Microsoft Excel (Version 16.79.1) and R (Version 4.1.0). Descriptive statistics were generated for the study population. IRMCs were categorized as listed in Table 1 and absolute counts were calculated for each category. Incidence rates were derived from absolute counts per category and estimated exposure to risk in hours. Calculations were performed separately for each sex, and results presented as the mean ​± ​SD accordingly.

Chi-square tests for goodness-of-fit were used to examine the distribution of injuries, assessing lateralization (right vs. left) and comparing frequencies across aggregated body regions (head and neck, upper limb, trunk, and lower limb). To compare incidence rates between sexes, the Incidence Rate Ratio (IRR) was calculated, and a 95 ​% confidence interval.

## 3 Results

### 3.1 Descriptive data

Eligibility was confirmed for all 69 athletes included in the study. Anthropometric data for the athletes is shown in Table 2.

**Table 2:** Anthropometric data of investigated athletes.

Sex	N	Age (years)	Height (cm)	Weight (kg)	BMI (kg/m2)
Female	30	19.6 ​± ​4.7	166.8 ​± ​6.3	60.3 ​± ​6.5	21.5 ​± ​2.0
Male	39	19.5 ​± ​3.9	177.2 ​± ​6.5	73.0 ​± ​7.8	22.6 ​± ​1.8

*Note. M* ± *SD*, BMI = Body-Mass-Index.

#### 3.1.1 Exposure to risk

Female athletes started practicing STSS at the national training center at 11.2 ​± ​5.1 years of age, while male athletes started at 10.5 ​± ​3.6 years. Duration of a complete STSS career for female athletes was 10.2 ​± ​4.8 years (1.4–20.7, n ​= ​30) and 11.4 ​± ​4.0 years (3.3–19.6, n ​= ​39) for male athletes. A total of 759 years in STSS were documented for all athletes.

Depending on their age, athletes trained between 5 and 35 ​h per week. The scope of training started at 5 ​h, divided into two sessions a week, at the athletes’ age of 10 years. It then constantly increased up to 30 ​h per week at the age of 21 and then plateaued for the rest of the career. Female athletes trained an average of 21.3 ​h per week, distributed over 8.2 training sessions. Assuming 40 weeks of training in a year, this amounted to 851 ​h in 328 training sessions per year, and an average of 8697 ​± ​4106 ​h of exposure to risk throughout an average career. In total, approximately 260,907 exposure hours were documented for all female athletes. Male athletes trained an average of 25.0 ​h per week, distributed over 9.5 training sessions. Assuming 40 training weeks per year, this corresponded to 999 ​h in 379 training sessions per year, and an average of 11,208 ​± ​4220 ​h of exposure to risk throughout the average career. In total, approximately 437,102 exposure hours were documented for all male athletes combined.

#### 3.1.2 Injury incidence

Of the 69 athletes whose medical histories were evaluated, 65 athletes (94.0 ​%) experienced at least one IRMC. The average age at the first IRMC was 17.0 ​± ​4.0 years with 17.5 ​± ​5.1 years for female, and 16.6 ​± ​3.0 years for male athletes. On average, the first IRMC occurred after 6.4 ​± ​3.5 years in STSS, with female athletes averaging 6.3 ​± ​3.6 years and male athletes 6.5 ​± ​3.5 years in the sport. During their careers, STSS athletes had a median of three IRMCs (four for males and three for females). Female athletes suffered an average of 4.2 ​± ​3.6 IRMCs, while male athletes had an average of 4.6 ​± ​3.3. Statistical analysis showed no significant difference in the mean or median number of IRMCs per athlete's career between sexes (t-test *p* ​= ​0.605; Mann–Whitney U test *p* ​= ​0.634). In total, of 371 IRMCs were documented, with 227 among males and 144 among females. This corresponds to an overall incidence of 0.53 IRMCs per 1000 ​h of exposure to risk (0.52 per 1000 ​h for males and 0.55 per 1000 ​h for females). Although the incidence rate for female athletes was approximately 4.0 ​% higher than for male athletes (Incidence Rate Ratio ​= ​1.038), this difference was not statistically significant.

#### 3.1.3 Circumstances of injury

64.0 ​% of the documented orthopedic-traumatological injuries were *directly* associated with STSS training or competition. Further analyses revealed that 45.0 ​% of IRMCs occurred during on-ice training, 30.0 ​% during off-ice training, and 25.0 ​% during on-ice competitions. 0.8 ​% of cases were *indirectly* associated with STSS training or competition e.g., on the way to or from training. In 35.2 ​% of cases, the association of injury with STSS was undocumented. When comparing on- and off-ice injuries between male and female athletes, no statistically significant difference was found (χ^2^ ​= ​0.082, *p* ​= ​0.774).

#### 3.1.4 Pathomechanism and mode of onset of injury

The pathomechanism was either *acute,* leading to a *sudden onset* of symptoms, or *repetitive,* leading to a *gradual* or *sudden onset* of symptoms. In 56.1 ​% of documented IRMCs, an acute pathomechanism caused the injury; 21.3 ​% were attributed to a repetitive pathomechanism, and 22.6 ​% went undocumented in this respect. A sudden onset of symptoms characterized 56.6 ​% of IRMCs, 15.1 ​% were described with a gradual onset and 28.3 ​% had no documentation regarding onset of symptoms. For IRMCs with a sudden onset, 25.7 ​% of injuries were secondary to falls to the ice or collision with the rink barriers. Subsequent injuries, exacerbations and injury severity could not be classified due to lack of data.

#### 3.1.5 Localization of injury

An analysis of the *body regions and area of injury* yielded the following frequencies: head and neck (6.3 ​%), upper limb (11.7 ​%), trunk (12.3 ​%), lower limb: 69.8 ​%. A chi-square test for goodness-of-fit revealed a statistically significant difference in injury distribution across these regions (χ^2^ ​= ​395.28, *p* ​< ​0.001), with the lower limb being the most frequently affected region. Categories 'unspecified' and 'multiple' had no recorded injuries in this dataset. Additionally, a comparison of injury distribution across body regions between male and female athletes showed no statistically significant difference (χ^2^ ​= ​1.85, *p* ​= ​0.605).

**Fig. 1:**
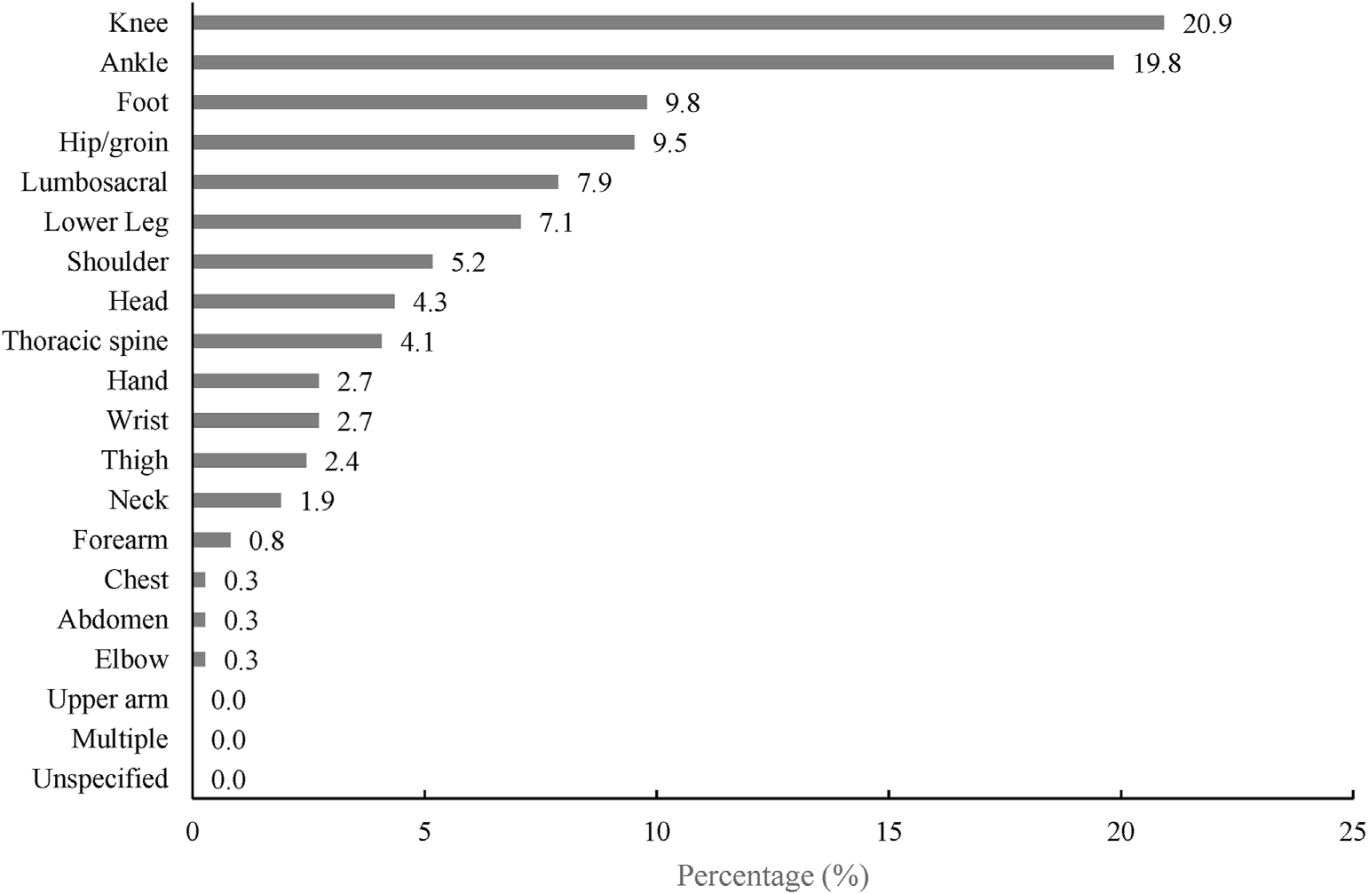
Anatomical distribution of orthopedic and traumatological injuries requiring medical consultation. ***Note.****N* ​= ​369. Chest ​= ​includes internal organs, Abdomen ​= ​includes internal organs, Multiple ​= ​Injury crossing two or more body regions.

Within the lower limb, the knee (20.9 ​%) and ankle (19.8 ​%) were the most frequently affected areas, followed by the foot (9.8 ​%), hip/groin (9.5 ​%), lumbosacral spine (7.9 ​%), and lower leg (7.5 ​%). Among upper limb injuries, the shoulder was involved in 5.2 ​% of IRMCs, while head injuries accounted for 4.3 ​% and thoracic spine injuries for 4.1 ​%. The distribution of IRMCs by anatomy is summarized in Fig. 1.

In addition to the analysis recommended by the IOC, lateralization of injuries was investigated . Injuries could be classified as either left-sided, right-sided, bilateral, or, in case of spinal injuries, be classified as midsagittal. While injuries were slightly more frequent on the right side (38.0 ​% right vs. 31.5 ​% left), the chi-square test did not show a statistically significant tendency toward right-sided injuries (χ^2^ ​= ​2.23, *p* ​= ​0.135). Midsagittal injuries accounted for 15.9 ​%, whereas 7.8 ​% of injuries were described as bilateral groin and/or hip pain. Lateralization was not documented in 6.7 ​% of IRMCs. Furthermore, when comparing male and female athletes, there was no statistically significant difference in the distribution of injury lateralization between genders (χ^2^ ​= ​2.45, p ​= ​0.654).

At least one anatomical malalignment was diagnosed in 17.0 ​% of the athletes, with 65.0 ​% affecting the foot, 24.0 ​% affecting the hip, and 11.0 ​% affecting the knee.

#### 3.1.6 Tissue and pathology types for injury

The analysis of the affected tissue, according to Table 5 of the IOCs’ consensus statement, revealed the following distribution: bony tissue (29.9 ​%), unspecified (26.1 ​%), ligaments and joint capsules (17.0 ​%), and muscle and tendons (12.1 ​%). Neurological (0.8 ​%) and vascular (0.5 ​%) systems were rarely affected [9].

Injuries involving a joint sprain with partial or complete rupture of the involved ligament structures and/or an episode of acute instability accounted for 16.3 ​% with an incidence of 0.86 per 10,000 ​h of exposure to risk. Additionally, 30.9 ​% of all IRMCs were classified as *unspecified* in this aspect. Further information on tissue and pathology types for injuries, according to the IOCs’ consensus statement, is displayed in Table 3 [9].

**Table 3:** Tissue and pathology types for injuries in German National STSS athletes.

OSIICS	SMDCS	Characteristics $\omega_{k}$	Absolute Frequency $n_{k}$	Percentage [%]	Incidence/10,000 ​h
M	10.07–10.09	Muscle injury	13	3.5	0.186
H	10.24	Muscle contusion	5	1.4	0.072
Y	10.36	Compartment syndrome	0	0.0	0.000
T	10.28–10.29	Tendinopathy	26	7.0	0.372
R	10.09	Tendon rupture	3	0.8	0.043
N	20.40	Brain/spinal cord injury	2	0.5	0.029
N	20.39, 20.41–42	Peripheral nerve injury	1	0.3	0.014
F	30.13–30, 16. 30.19	Fracture	28	7.6	0.401
S	30.18, 30.32	Bone stress injury	27	7.3	0.387
J	30.24	Bone contusion	29	7.9	0.415
E	30.35	Avascular necrosis	2	0.5	0.029
G	30.20	Physis injury	0	0.0	0.000
C	40.17, 40.20, 40.37	Cartilage injury	12	3.3	0.172
A	40.33–40.34	Arthritis	0	0.0	0.000
Q	40.22, 40.34	Synovitis/Capsulitis	1	0.3	0.014
B	40.31	Bursitis	3	0.8	0.043
L Or D	50.01–50.11	Joint sprain^a^	60	16.3	0.860
U	50.12	Chronic instability	2	0.5	0.029
V	60.24	Contusion superficial	17	4.6	0.244
K	60.25	Laceration	16	4.3	0.229
I	60.26–60.27	Abrasion	6	1.6	0.086
V	70.45	Vascular trauma	2	0.5	0.029
O	80.46	Other internal organs	0	0.0	0.000
P Or Z	00.00	Nonspecific (injury without tissue type specified)	114	30.9	1.633

***Note.****N* ​= ​369. OSIICS: Orchard Sports Injury and Illness Classification System, SMDCS: Sports Medicine Diagnostic Coding System, modified from table 5 of the IOC consensus statement on methods for recording and reporting of epidemiological data on injury and illness in sports 2020. *The Orthopaedic Journal of Sports Medicine*, *8*(2), 325967120902908. https://doi.org/10.1177/2325967120902908.

a Joint sprain: partial or complete rupture of ligamentous structures and/or an episode of acute instability.

## 4 Discussion

STSS presents a unique set of challenges to athletes due to its high-intensity nature and close proximity among competitors during races. This retrospective analysis provides valuable insights into orthopedic and traumatological injuries among German national STSS athletes, offering comparisons to previous studies and analyzing injury patterns based on the standardized injury surveillance system published by the IOC.

### 4.1 Key results

The average career lasted 10.9 ​± ​4.4 years. During this time, an athlete accumulated 10,116 ​± ​4326 ​h of exposure to risk. The first IRMC occurred, on average, after 6.4 ​± ​3.5 years in STSS at an average age of 17.0 ​± ​4.0 years. In general, the incidence of IRMCs was estimated to be 0.53 for every 1000 ​h of exposure to risk. Analysis of the anatomical distribution of injuries revealed that 69.8 ​% of cases were related to the lower extremity, with the knee (20.8 ​%) and the ankle (19.8 ​%) being the most affected. A chi-square test for goodness-of-fit confirmed a statistically significant difference in injury distribution across body regions (χ^2^ ​= ​395.28, *p* ​< ​0.001), indicating that lower extremity injuries were disproportionately high compared to other regions. Although injuries were slightly more frequent on the right side (38.0 ​% right vs. 31.5 ​% left), the chi-square test did not indicate a statistically lateralization (χ^2^ ​= ​2.23, *p* ​= ​0.135). Joint injuries involving partial or complete rupture of ligamentous structures and/or episodes of acute instability accounted for 16.3 ​% (incidence of 0.86 for 10,000 ​h of exposure to risk), making it the most common type of injury in the study. In total, 45.0 ​% of all IRMCs occurred during on-ice training, 30.0 ​% during off-ice training, and 25.0 ​% during competitions.

### 4.2 Interpretation

#### 4.2.1 Injury incidence

The average career duration of 10.9 ​± ​4.4 years and accumulated hours of exposure to risk of 10,116 ​± ​4326 ​h in training highlight the prolonged and intensive nature of STSS participation. Male athletes had longer careers overall, with greater total hours of exposure both over their entire career and on an annual basis. The incidence of IRMCs averaged 0.53 per 1000 ​h of exposure for both sexes combined, with a slightly higher, though not statistically significant, incidence observed in female athletes (0.55 for females vs. 0.52 for males). With an estimated exposure to risk of 1000 ​h per year, we calculated the annual probability of an orthopedic-traumatological injury to be 53.0 ​% (female: 55.0 ​%; male: 52.0 ​%). In comparison, Brownlow and McCaig (2021) reported a significantly higher injury incidence of 4.4 per 1000 ​h of exposure to risk in a Great Britain elite STSS training group. The drastic difference in values could be due to different methods of documenting exposure to risk and/or injuries, such as the incorporation of self-reported injuries. Quinn et al. [1] calculated a probability of 64.2 ​% for STSS athletes to experience at least one injury during a season. Dittmer [2] surveyed 100 STSS athletes over a period of 18 months. According to their findings, an athlete suffered 0.38 injuries annually. For injury incidence in competition, Gallo-Vallejo et al. [6] found that STSS athletes competing in the 27th Winter Sports Universiade 2015 in Granada incurred an injury after 9.8 ​h of exposure to risk. Chiado Pirat et al. [4] found that 0.9 ​% of all STSS Athletes participating in the 2006 Olympic Winter Games were injured. In contrast, Engebretsen et al. (2020) reported 27.8 ​% of male athletes and 5.0 ​% of female athletes got injured in the 2010 Olympic Winter Games. For both studies, exposure to risk was defined by the participation in the multisport event and was not linked to a specific number of hours in the sport.

In conclusion, the reported injury incidence rates vary greatly and need to be distinguished between training and competition. The variation in reported numbers highlights the importance of standardization. The current study suggests an incidence of 53.0 ​% for IRMCs in the orthopedic-traumatological field in one year of professional STSS.

#### 4.2.2 Circumstances of injuries

The analyses revealed that 45.0 ​% of IRMCs occurred during on-ice training, 30.0 ​% during off-ice training, and 25.0 ​% during on-ice competitions, with no statistically significant differences observed between these categories. This is consistent with the findings of previous studies showing that 80.0 ​% of injuries occurred during training and 20.0 ​% during competitions [1,5]. Information on exposure to risk during competition was lacking in these two previous studies as well as in the present one. The incidence of injury during competition could therefore not be elicited. Given the total annual exposure to risk in comparison to the shorter exposure to risk during competition, we assume the incidence of injury during competition to be higher than that for training.

#### 4.2.3 Mode of onset of injury

Within the present study, 56.6 ​% of the injuries had a sudden onset, whereas 15.1 ​% had a gradual onset. Injuries with sudden onset were further categorized according to the IOC's consensus statement.

The largest proportion (25.7 ​%) of injuries with sudden onset resulted from falls or collisions with the rink barrier or through the athlete's own skating blades. Additionally, 20.0 ​% of injuries were incurred through collisions between athletes. 16.0 ​% of acute injuries occurred without external influence (e.g., ankle sprain). In 34.0 ​% of cases, the mechanism of injury was unspecified.

To our knowledge, this is the first study to classify orthopedic-traumatological injuries in STSS according to this aspect of the IOCs consensus statement. We found some overlap of our findings with those from Palmer–Green et al. [10] and Dittmer [2], showing that injuries incurred from collisions and falls account for 25.0 ​% of all injuries observed, as we did at 25.7 ​%. Dittmer [2] found that *contact with other athletes* specifically was responsible for 9.0 ​% of injuries, compared to the 20.0 ​% observed in the present study. Again, neither the discrepancies nor the similarities to our findings can be accurately interpreted, owing to the differences in surveillance and documentation.

#### 4.2.4 Body regions and areas for injury

Injuries to the lower extremities were observed with the greatest frequency, accounting for 69.8 ​% of all injuries in the current study. A chi-square test for goodness-of-fit revealed a statistically significant difference in the distribution of injuries across body regions (χ^2^ ​= ​395.28, *p* ​< ​0.001), confirming that lower extremity injuries were disproportionately high compared to other regions. Additional analysis tested for differences in injury distribution between sexes, revealing no statistically significant variation. This finding aligns with previous studies by Dittmer [2], who reported 62.0 ​%, and Brownlow and McCaig (2021), who also found lower extremity and lumbar spine injuries to be the most prevalent. Within the lower extremity, knee injuries were the most common, representing 20.9 ​% of all injuries, comparable to the 19.0 ​% reported by Palmer–Green et al. [10] and 23.4 ​% by Quinn et al. [1]. Ankle injuries had the second-highest prevalence in the present study at 19.8 ​%, similar to the 14.4 ​% reported by Quinn et al. [1]. These findings underscore the importance of targeted measures to address vulnerabilities for the knee and the ankle.

Other injuries were distributed as follows: trunk (12.3 ​%), upper extremities (11.7 ​%) and head and neck (6.3 ​%). The prevalence of injuries to the trunk in the present study is higher than the 3.0 ​% found by Dittmer [2], but similar to the 19.0 ​% found by Palmer–Green et al. [10], and the 13.0 ​% found by Quinn et al. [1]. Dittmer [2] found a lower incidence of upper extremity injuries than in the present study (11.7 ​% vs. 21.0 ​%), while Palmer–Green et al. [10] and Quinn et al. [1] did not provide data on this category. Head injuries accounted for 6.3 ​% of the injuries in the present study, fewer than the 14.0 ​% reported by Dittmer [2] but comparable with the 5.4 ​% calculated by Quinn et al. [1]. In the analysis of specific affected body regions, all regions except the upper arm were affected by health issues.

Our findings also indicated a slight predominance of right-sided injuries compared to the left, though this difference was not statistically significant. Increased loading on the right lower extremity during the left turns inherent to STSS has been documented Hesford et al., 2012 [11], which may partially account for this trend. To our knowledge, no other study has specifically examined lateralization of injuries in STSS athletes. Overall, our observations regarding the distribution of injuries by body region are largely consistent with findings from previous literature.

#### 4.2.5 Tissue and pathology types for injury

Firstly, attention needs to be drawn to the 30.9 ​% of all IRMCs that were *unspecified* in this regard, as seen in Table 3. This may be the result of the treating physician not being familiar with the classification system. For example, a medical record might report “groin pain to the left side after fall during competition on the weekend", leaving the exact tissue and pathology types of the injury unspecified. The analysis of the diagnoses that did provide precise information on tissue and pathology types for injuries reveals that ankle sprains and contusions of the knee, caused by impact trauma, were the most common injuries documented. According to Dittmer [2], the most common types of injuries in descending order are leg lacerations, ankle ligament injuries, knee contusions, lower leg fractures, chin lacerations, and shoulder contusions. Lacerations occurred less frequently in the present study, accounting for 4.3 ​% of injuries, compared to previous research by Dittmer [2], Snouse et al. [8], and Quinn et al. [1], where they accounted for approximately 10.0 ​% of injuries. One possible explanation for the lower proportion of lacerations compared to previous studies could be the development of cut-resistant protective equipment. Although, no other studies in STSS use the same IOC categorization for tissue and pathology types of injuries, our findings appear to be consistent with previous studies.

### 4.3 Limitations

A fundamental limitation of the analysis is its retrospective design, with a lack of standardized documentation by treating physicians. Approximately 10.0 ​% of cases, had incomplete records for one or more investigated categories, including diagnosis. In addition, follow-up with athletes was outside the scope of the study.

While the average number of weekly training hours was documented in each athlete's annual medical examination, this average did not account for variations throughout the year, nor did it specify training modalities (on-ice, off-ice training, etc.). Additionally, time spent in competitions was not recorded. The analysis was limited to orthopedic and traumatological injuries and did not include internal illnesses. The IOC recommends documentation of the latter, referencing the fact that these are interdependent conditions [9]. Furthermore, only 5.0 ​% of IRMCs included information on *time loss* and the rehabilitative process. Information which is necessary to quantify injury severity.

In summary, the retrospective design constrained the study's ability to capture crucial details, including competition-related risk exposure, injury context, internal illnesses, and post-injury recovery time.

### 4.4 Conclusion

In conclusion, this study sheds light on the orthopedic and traumatological injuries within German national STSS, revealing insights into injury incidence, circumstances, modes of onset, affected body regions, and tissue/pathology types. The findings underscore the demanding nature of STSS, with a notable prevalence of lower extremity injuries, particularly to the knee and ankle. While the retrospective design and limitations in documentation posed challenges, the study provides valuable data for understanding injury patterns in STSS athletes. Moving forward, efforts towards standardization in injury surveillance and documentation methodologies, including reports of incidences not just distributions of injuries, are crucial for better comparability across studies and, ultimately, for the development of targeted injury prevention strategies and interventions in the sport.

## 5 Practical implications

• The identified patterns of injuries partly confirmed previous studies while also providing new information, such as the timing of injury during athletes' careers and the lateralization of injuries.
• The retrospective implementation of the IOC's recommendations on methods for recording and reporting epidemiological data on injury and illness in sports, as published by the IOC in 2020, proved to be rather difficult since relevant information was often missing.
• In clinical practice, a balance must be struck between reasonable documentation efforts and capturing all relevant aspects of health problems. For instance, Appendices 2A and 2B of the consensus statement might be used to this end.
• Further studies are needed to identify and understand mechanisms underlying injuries in STSS.
• The implementation of technology, such as wearable tracking devices or apps for self-reporting of medical conditions, may contribute to help capture data more easily, identify risk factors of health problems, improve prevention efforts, and ultimately promote the health and performance of athletes.

## Confirmation of ethical compliance

The study complied with the ethical standards and with the Helsinki Declaration of 1975, as revised in 2008, and was approved by the ethics committee of the Technical University of Dresden (BO-EK-185032021). Informed consent was obtained from the study participants.

## Credit authorship contribution statement

Conceptualization: Heidrun Beck, Stefan Zwingenberger, Jan Ziegler.

Methodology: Jan Ziegler.

Validation: Heidrun Beck, Stefan Zwingenberger.

Formal analysis: Jan Ziegler.

Data curation: Jan Ziegler.

Writing—original draft preparation: Jan Ziegler.

Writing—review and editing: Heidrun Beck, Roland L. Bell, Dominik Matzner, Xinggui Tian, Stefan Zwingenberger, Jan Ziegler.

Visualization: Jan Ziegler.

Project administration: Jan Ziegler.

Funding acquisition: Jan Ziegler.

## Data availability statement

The data are not publicly available due to privacy regulations related to data that can be traced back to an individual participant.

## Declaration of generative AI and AI-assisted technologies in the writing process

During the preparation of this work the author(s) used [ChatGPT 3.5/Open AI] to [improve grammar and spelling]. After using this tool/service, the author(s) reviewed and edited the content as needed and take(s) full responsibility for the content of the publication.

## Funding information

This work was funded by the Technical University of Dresden through a transformative agreement between the Saxon State and University Library (SLUB) of TU Dresden and Elsevier, which covered the article processing charge (APC). The funding organization was not involved in the collection of data, their analysis and interpretation, and in the right to approve or disapprove publication of the finished manuscript. Other than that, this research did not receive any specific grant from funding agencies in the public, commercial, or not-for-profit sectors.

## Declaration of competing interest

None.
