# Supplementary material for: Injuries in German national short-track speed skating athletes
Source: JSAMS Plus. 2025 Jan 2;5:100080. doi: 10.1016/j.jsampl.2024.100080 (PMC13008447; doi:10.1016/j.jsampl.2024.100080)
Supplement: Multimedia component 1 [file mmc1.docx]

**Supplementary material**

**Table 1:** Previous studies on health issues in STSS athletes.

| Authors | *N* | Design | (Age) (Level) | Timespan observed | Investigations |
| --- | --- | --- | --- | --- | --- |
| (Brownlow & Mc Caig, 2021) | 9 m  6 f | Retrospective | (17-33)  (Great Britain-National-team) | 24 months | Incidence of injuries and illness  Training and competition Time lost (“Time loss”)  Anatomical distribution of injury  Circumstances of fall or injury |
| (Chiadò Piat et al., 2010) | 110 | Retrospective | (N/A) (National-teams) | Olympic Winter Games 2006 | Incidence of injury |
| (Dittmer, 2015) | 56 m  44 f | Retrospective | (8-27)  (N/A) | 2002 - 2004  18 months | Anatomical distribution of injury  Most common injury  Cause of injury  Overuse injury  Anatomical malalignments |
| (Engebretsen et al., 2010) | 55 m  54 f | Prospective | (N/A) (National-teams) | 2010 Olympics | Incidence of injury |
| (Gallo-Vallejo et al., 2017) | 50 m  44 f | N/A | (N/A) (N/A) | “Granada Winter Sports Universiade”, 2015 | Incidence of injury  Anatomical distribution of injury |
| (Hillis, 2018) | N/A | Prospective | (7-12)  (N/A) | Season 2012/13  14 events | Incidence of injury |
| (Palmer-Green et al., 2014) | 7 m  4 f | Prospective | (N/A) (Great Britain National-team) | Season 2009/10 | Anatomical distribution of injury  Training and competition Time lost (“Time loss”)  Cause of injury |
| (Quinn et al., 2003) | 95 | Retrospective | (N/A) (US-elite athletes) | Season 1999 – 2000 | Anatomical distribution of injury  Type of injury  Circumstances of fall or injury |
| (Snouse et al., 1999) | 2120 | Prospective | (N/A) (N/A) | 1993 -1999  36,562 days of practice | Lacerations  Training and competition Time lost (“Time loss”) |

**Note:** N/A = not available; m = male; f = female.
